# Alteration of β-Adrenoceptor Signaling in Left Ventricle of Acute Phase Takotsubo Syndrome: a Human Study

**DOI:** 10.1038/s41598-018-31034-z

**Published:** 2018-08-24

**Authors:** Tomoya Nakano, Kenji Onoue, Yasuki Nakada, Hitoshi Nakagawa, Takuya Kumazawa, Tomoya Ueda, Taku Nishida, Tsunenari Soeda, Satoshi Okayama, Makoto Watanabe, Hiroyuki Kawata, Rika Kawakami, Manabu Horii, Hiroyuki Okura, Shiro Uemura, Kinta Hatakeyama, Yasuhiro Sakaguchi, Yoshihiko Saito

**Affiliations:** 1 First Department of Internal Medicine, Nara Medical University, Kashihara Nara, Japan; 2 Department of Diagnostic Pathology, Nara Medical University, Kashihara Nara, Japan; 3 Present Address: Department of Cardiology, Nara City Hospital, Nara, Japan; 4 Present Address: Department of Cardiology, Kawasaki Medical School, Kurashiki, Japan

## Abstract

Accumulating evidence indicates alteration of the β-adrenoceptor (AR), such as desensitization and subtype switching of its coupling G protein, plays a role in the protection against catecholamine toxicity in heart failure. However, in human takotsubo syndrome (TTS), which is associated with a surge of circulating catecholamine in the acute phase, there is no histologic evidence of β-AR alteration. The purpose of this study was to investigate the involvement of alteration of β-AR signaling in the mechanism of TTS development. Left ventricular (LV) biopsied samples from 26 patients with TTS, 19 with normal LV function, and 26 with dilated cardiomyopathy (DCM) were studied. G protein-coupled receptor kinase 2 (GRK2) and β-arrestin2, which initiate the alteration of β-AR signaling, were more abundantly expressed in the myocardium in acute-phase TTS than in those of DCM and normal control as indicated by immunohistochemistry. The percentage of cardiomyocytes that showed positive membrane staining for GRK2 and β-arrestin2 was also significantly higher in acute-phase TTS. Sequential biopsies in the recovery-phase for two patients with TTS revealed that membrane expression of GRK2 and β-arrestin2 faded over time. This study provided the first histologic evidence of the involvement of alteration of β-ARs in the development of TTS.

## Introduction

Takotsubo syndrome (TTS) is characterized by transient hypokinesis of the left ventricular (LV) apex and hyperkinesis of the LV base without coronary artery abnormality^1–5^. The LV dysfunction is generally reversible with favorable outcome^1,2,5,6^. Typically, postmenopausal women are susceptible to TTS, which is frequently triggered by intense emotional or a physical stress^2–9^. Several mechanisms of TTS have been proposed to date, including multivessel spasm, acute microvascular dysfunction, reactive oxygen species (ROS) production, and catecholamine cardiotoxicity^1,2,4,5,7,10–12^. In particular, cardiac sympathetic nervous system (SNS) activation has been implicated as an important trigger for developing TTS in studies that showed a significant elevation in plasma catecholamine in patients with acute-phase TTS as compared to those with acute myocardial infarction^1^, and the elevated catecholamine concentration normalized within a few days^1,13–15^. However, direct evidence of these mechanisms from human tissues was not obtained, nor has the concept been established yet.

In physiological condition, the SNS regulates both inotropic and chronotropic cardiac functions directly through noradrenaline released from its nerve endings and indirectly through adrenaline released from the adrenal gland via the β-adrenoceptor (AR) in response to body demands. However, in pathological conditions such as heart failure, plasma catecholamine levels are elevated to supraphysiological levels and the subsequent overactivation of β-AR/protein kinase A (PKA) signaling results in various degrees of cardiomyocyte necrosis and apoptosis, termed “catecholamine toxicity”^15–18^. Over the past two decades, accumulating evidence has shown that the β-AR signaling system itself has several mechanisms of alteration for the protection from catecholamine toxicities, such as receptor desensitization and subtype switching of its coupling G protein from stimulatory Gαs to inhibitory Gαi, which protects from overactivation of PKA, resulting in reduced pumping function^16–21^. These mechanisms are initiated by the overexpression of G protein-coupled receptor kinase 2 (GRK2) and β-arrestin2, and their subsequent translocation to the cell membrane^18,22^.

We hypothesized that alteration of β-AR signaling is involved in the mechanism of TTS development. To confirm this hypothesis, we examined the expression profiles of GRK2 and β-arrestin2 in human biopsied samples obtained from the postero-apical portion of the LV, where wall motion was markedly reduced in the acute phase.

## Results

### Baseline Characteristics

Baseline clinical characteristics of the three patient groups, including cardiovascular risk factors and medications on admission, are presented in Table 1. Patients in the TTS group were significantly older, with a higher proportion of females and smaller body constitution than those in the normal control (NC) and dilated cardiomyopathy (DCM) groups. Medications on admission were not significantly different among the three groups.

**Table 1:** Patient Characteristics.

	Normal control (n = 19)	Takotsubo syndrome (n = 26)	Dilated cardiomyopathy (n = 26)	P-value
Age (years)	54.3 ± 19.2	72.1 ± 11.9	54.7 ± 13.0	<0.001
Female sex, number (%)	2 (11.1)	22 (84.6)	8 (30.8)	<0.001
Height (cm)	166.6 ± 8.5	154.1 ± 9.6	164.6 ± 7.3	<0.001
Body Weight (kg)	63.4 ± 11.3	51.0 ± 10.7	64.0 ± 13.8	<0.001
Previous History				
Hypertension, number (%)	5 (26.3)	14 (53.9)	15 (57.7)	0.09
Diabetes mellitus, number (%)	3 (15.8)	4 (15.4)	5 (19.2)	0.92
Dyslipidemia, number (%)	6 (31.6)	5 (19.2)	8 (30.8)	0.55
Medical treatment on admission				
ACE-Is/ARBs, number (%)	4 (21.1)	6 (23.1)	7 (26.9)	0.89
β-receptor blocker, number (%)	0 (0.0)	0 (0.0)	0 (0.0)	
Aldosterone blocker, number (%)	1 (5.3)	1 (3.9)	5 (19.2)	0.13

Values are mean ± standard deviation or number of patients (%) as appropriate. ACE-Is/ARBs: angiotensin-converting enzyme inhibitors/angiotensin II receptor blockers.

Cardiac parameters, obtained by left ventriculography, are presented in Table 2. LV end systolic volume index and LV ejection fraction of the TTS group were between those of the NC and DCM groups. LV functional recovery was verified by echocardiography in all patients before discharge (for details, see Supplementary Table S1).

**Table 2:** Clinical Characteristics.

	Normal control (n = 19)	Takotsubo syndrome (n = 26)	Dilated cardiomyopathy (n = 26)	P-value
Left ventriculography data				
LVEDV (mL/m^2^)^*^	72.8 ± 22.0	78.3 ± 17.1	122.0 ± 33.1	<0.001
LVESV (mL/m^2^)^*^	26.1 ± 10.3	41.3 ± 16.6	81.9 ± 27.0	<0.001
LVEF (%)	64.0 ± 9.4	47.8 ± 14.0	33.2 ± 8.9	<0.001
Laboratory data on admission				
Uric acid (mg/dL)	6.4 ± 1.9	4.9 ± 1.3	7.0 ± 2.4	0.002
eGFR (mL/min/1.73 m^2^)^†^	73.1 ± 24.7	74.3 ± 20.9	73.7 ± 25.4	0.72
Corrected serum calcium (mg/dL)	9.4 ± 0.3	8.9 ± 0.4	9.2 ± 0.4	0.006
BNP (pg/mL)	66.2 ± 8.4	489.7 ± 406.6	678.5 ± 932.4	<0.001
PRA (ng/mL/hr)	2.5 ± 2.8	3.2 ± 6.2	4.3 ± 6.8	0.29
PAC (pg/mL)	154.5 ± 109.5	121.5 ± 127.6	145.1 ± 156.8	0.24
Plasma adrenaline (pg/mL)^‡§^	n/a	78.0 (38.0–268.7)	n/a	
Plasma noradrenaline (pg/mL)^‡¶^	n/a	671.0 (265.5–1169.5)	n/a	

Values are mean ± standard deviation or median (25^th^–75^th^ percentile) as appropriate. *Left ventricular end-diastolic volume (LVEDV) index (LVEDVI = LVEDV/body surface area (BSA)) and left ventricular end-systolic volume (LVESV) index (LVESVI = LVESV/BSA) were calculated by means of the area–length method. ^**†**^Estimated glomerular filtration rate (eGFR) was calculated according to the published equation for Japanese subjects: 194 × serum creatinine^−1.094^ × age^−0.287^ × (0.739 for women). ^**‡**^Plasma adrenaline and noradrenaline were measured within 1.1 ± 1.0 days after admission in 12 patients of the TTS group. ^**§**^The normal range of plasma adrenaline is 0–100 pg/mL. ^**¶**^The normal range of plasma noradrenaline is 100–450 pg/mL. LVEF: left ventricular ejection fraction, BNP: brain natriuretic peptide, PRA: plasma renin activity, PAC: plasma aldosterone concentration.

Laboratory data on admission of the three groups are presented in Table 2. Brain natriuretic peptide (BNP) level on admission in the TTS group was elevated to 489.7 ± 406.6 pg/mL, which dropped down markedly to 71.4 ± 60.9 pg/mL at discharge. In the TTS group, corrected serum calcium concentration on admission was 8.9 ± 0.4 mg/dL, which was significantly lower than that of the NC group, but returned to normal (9.3 ± 0.4 mg/dL) at discharge.

### Pathological Features

First, we assessed the LV biopsy specimens by hematoxylin-eosin (HE) and Masson’s trichrome (MT) stainings. The degree of interstitial fibrosis in the TTS group was significantly higher than that in the NC group, but similar to that in the DCM group (NC, 5.9 (3.9–10.8)%; TTS, 12.1 (7.8–21.1)%; DCM, 11.3 (4.0–16.0)%, P = 0.005) (for details, see Supplementary Fig. S1).

Next, we conducted immunohistochemical stainings of GRK2 and β-arrestin2, which exert negative inotropic effects through alterations of β-ARs and thus, protect against catecholamine cardiotoxicity. First of all, we performed western blotting analysis to confirm the specificity of the antibodies used for GRK2 and β-arrestin2 immunostainings. Both antibodies showed one specific band as presented in Supplementary Fig. S2.

**Figure 1:**
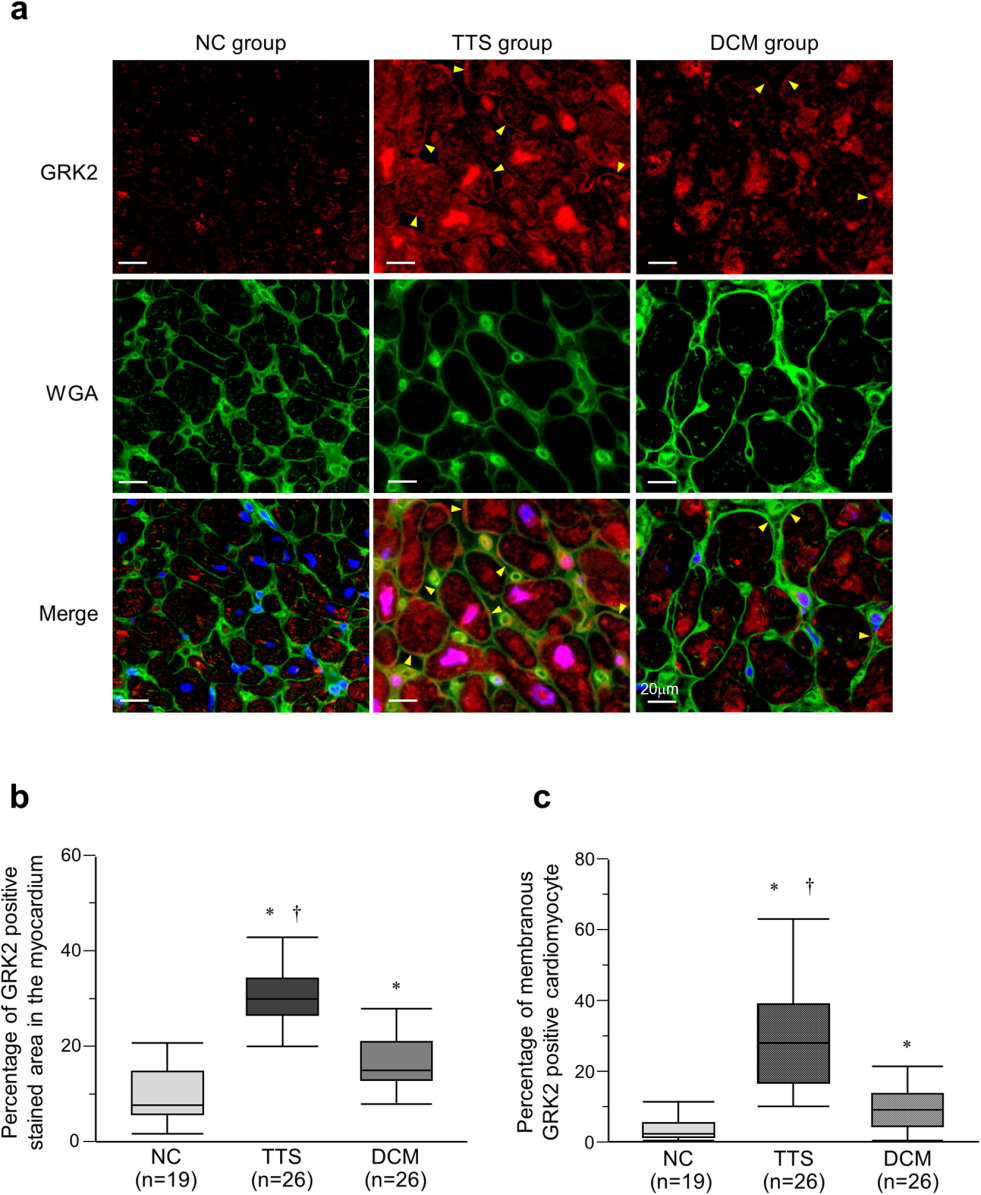
Localization of G protein-coupled receptor kinase 2 (GRK2). (**a**) Immunohistofluorescence staining for GRK2 using the specific antibody. GRK2 (red) in the takotsubo syndrome (TTS) and dilated cardiomyopathy (DCM) groups were observed not only in the cytoplasm but also on the cell membrane, which was confirmed by colocalization with wheat germ agglutinin (WGA) (green) (Arrowheads). Blue staining localized to 4′,6-diamidino-2-phenylindole (DAPI). (**b**) Quantification for GRK2 positive stained area in the myocardium. The box represents the 25^th^ and 75^th^ percentiles and the line the median value. Whiskers correspond to the 25^th^ percentile minus 1.5 times interquartile range (IQR) and to the 75^th^ percentile plus 1.5 IQR. (**c**) Quantification for membranous GRK2 positive cardiomyocyte. *P < 0.001 vs. the normal control (NC) group. ^†^P < 0.001 vs. the DCM group.

**Figure 2:**
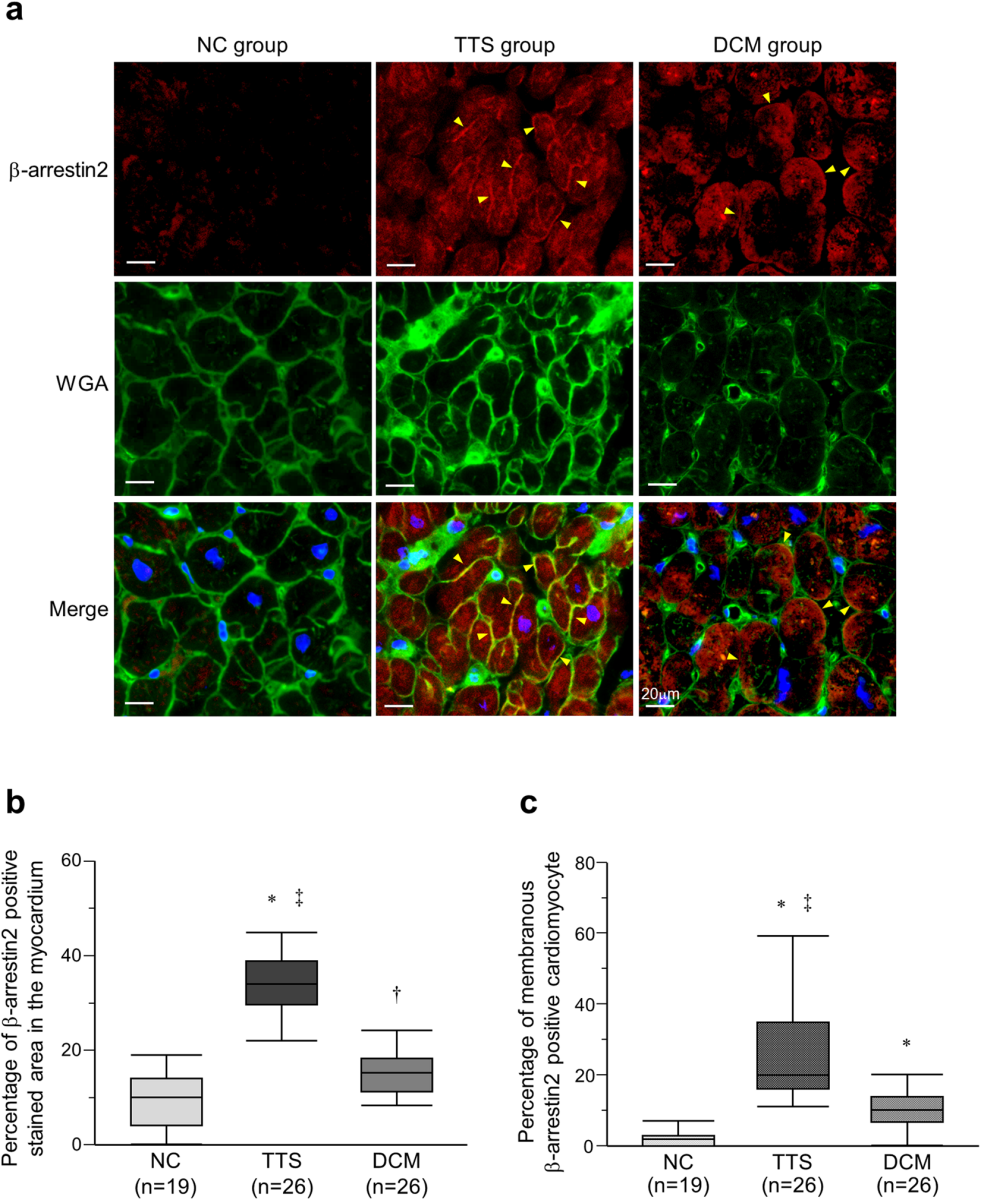
Localization of β-arrestin2. (**a**) Immunohistofluorescence staining for β-arrestin2 using the specific antibody. β-arrestin2 (red) in the takotsubo syndrome (TTS) and dilated cardiomyopathy (DCM) groups were observed not only in the cytoplasm but also on the cell membrane, which was confirmed by colocalization with wheat germ agglutinin (WGA) (green) (Arrowheads). Blue staining localized to 4′,6-diamidino-2-phenylindole (DAPI). (**b**) Quantification for β-arrestin2 positive stained area in the myocardium. The box represents the 25^th^ and 75^th^ percentiles and the line the median value. Whiskers correspond to the 25^th^ percentile minus 1.5 times interquartile range (IQR) and to the 75^th^ percentile plus 1.5 IQR. (**c**) Quantification for membranous β-arrestin2 positive cardiomyocyte. *P < 0.001 vs. the normal control (NC) group. ^†^P < 0.005 vs. the NC group. ^‡^P < 0.001 vs. the DCM group.

In the TTS and DCM groups, GRK2 signal was localized not only in the cytoplasm but also on the cell membrane, which was confirmed by colocalization with wheat germ agglutinin (WGA), whereas in the NC group, fluorescence was largely confined to the cytoplasm (Fig. 1a and Supplementary Fig. S3a). The GRK2-positive area in the myocardium was the greatest in the TTS group (NC, 8.0 (6.0–18.0)%; TTS, 30.0 (26.5–34.3)%; DCM, 15.0 (13.0–21.0)%, P < 0.001, Fig. 1b). Furthermore, the fraction of cardiomyocytes whose membrane was positively stained by GRK2 was the largest in the TTS group (NC, 2.0 (1.0–5.0)%; TTS, 28.0 (16.5–39.0)%; DCM, 8.5 (4.0–13.3)%, P < 0.001, Fig. 1c). Similarly, in the TTS and DCM groups, β-arrestin2 was localized both in the cytoplasm and on the cell membrane, where it colocalized with WGA (Fig. 2a and Supplementary Fig. S3b). The β-arrestin2-positive area in the myocardium was the largest in the TTS group (NC, 10.0 (4.0–14.0)%; TTS, 34.0 (29.8–39.0)%; DCM, 15.0 (11.0–18.0)%, P < 0.001, Fig. 2b). The percentage of cardiomyocytes with β-arrestin2-positive membranes was significantly larger in the TTS group than in the NC and DCM groups (NC, 2.0 (0.0–3.0)%; TTS, 20.0 (16.0–34.5)%; DCM, 10.0 (6.5–13.5)%, P < 0.001, Fig. 2c).

**Figure 3:**
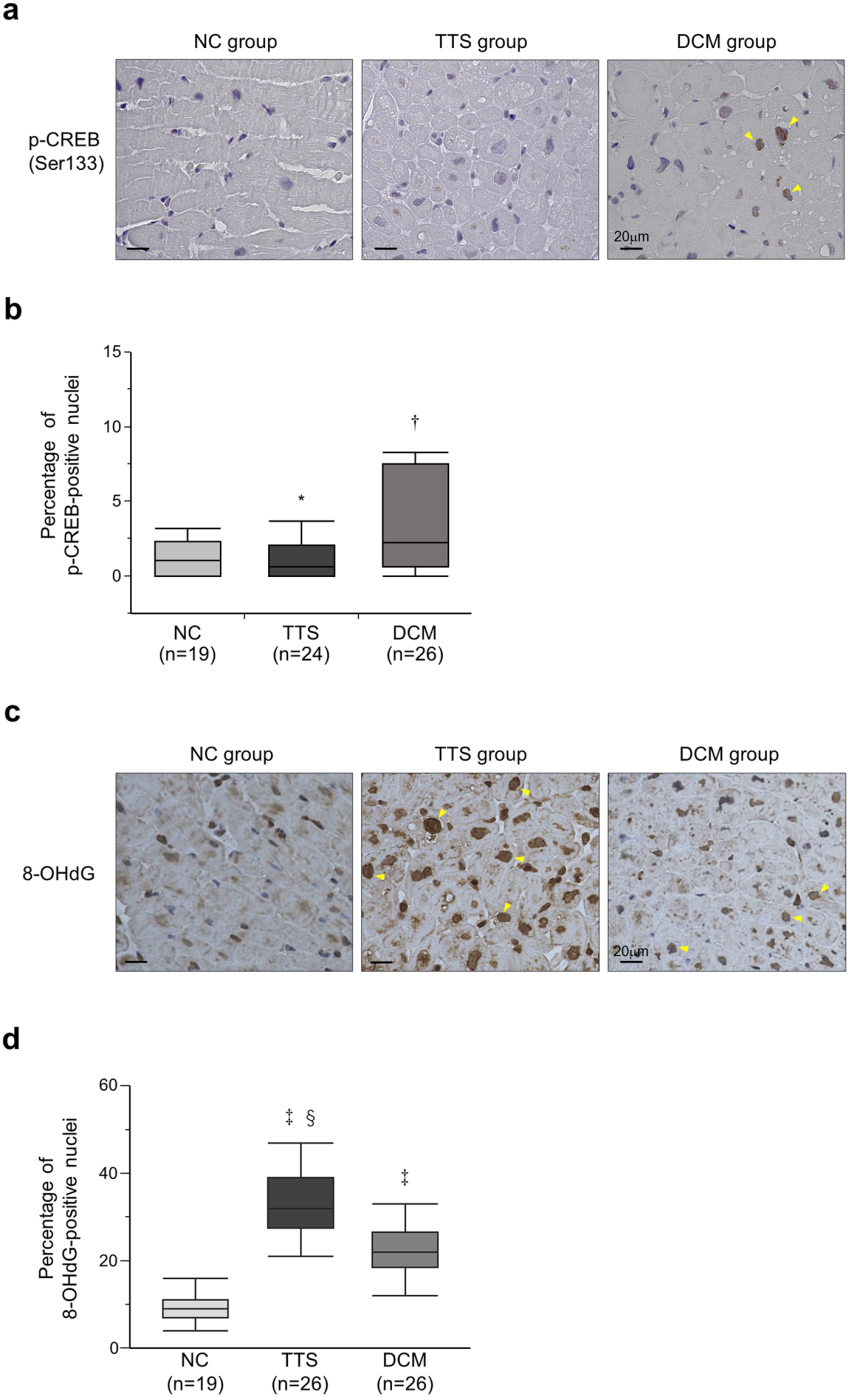
Localization of phosphorylated cyclic-AMP response element binding protein at Ser133 (pCREB (Ser133)) and 8-hydroxy-2′-deoxyguanosine (8-OHdG). (**a**) Immunohistochemical staining for pCREB, visualized by diaminobenzidine (brown) in cardiomyocyte (Arrowheads). (**b**) Quantification as the percentage of pCREB positively stained nuclei in total cardiomyocyte nuclei. (**c**) Immunohistochemical staining for 8-OHdG visualized by diaminobenzidine in cardiomyocyte (Arrowheads). (**d**) Quantification as the percentage of 8-OHdG positively stained nuclei in total cardiomyocyte nuclei. The box represents the 25^th^ and 75^th^ percentiles and the line the median value. Whiskers correspond to the 25^th^ percentile minus 1.5 times interquartile range (IQR) and to the 75^th^ percentile plus 1.5 IQR. *P < 0.05 vs. the dilated cardiomyopathy (DCM) group. ^†^P < 0.05 vs. the normal control (NC) group. ^‡^P < 0.001 vs. the NC group. ^§^P < 0.001 vs. the DCM group. TTS: takotsubo syndrome.

Immunostaining of phosphorylated cyclic-AMP response element binding protein (pCREB) was performed to evaluate β-AR/PKA signaling (Fig. 3a,b). The fraction of pCREB-positive cardiomyocyte nuclei in the TTS group was similar to that in the NC group, although it was significantly higher in the DCM group than in the NC and TTS groups (NC, 1.1 (0.0–2.3)%; TTS, 0.6 (0.0–2.1)%; DCM, 2.3 (0.7–7.5)%, P = 0.04, Fig. 3a,b). We also studied immunestaining of 8-hydroxy-2′-deoxyguanosine (8-OHdG) to examine ROS production. The fraction of 8-OHdG-positive cardiomyocyte nuclei was significantly higher in the TTS group than in the NC and DCM groups (NC, 9.0 (7.0–11.0)%; TTS, 32.0 (27.5–39.0)%; DCM, 22.0 (18.5–26.5)%, P < 0.001, Fig. 3c,d).

**Figure 4:**
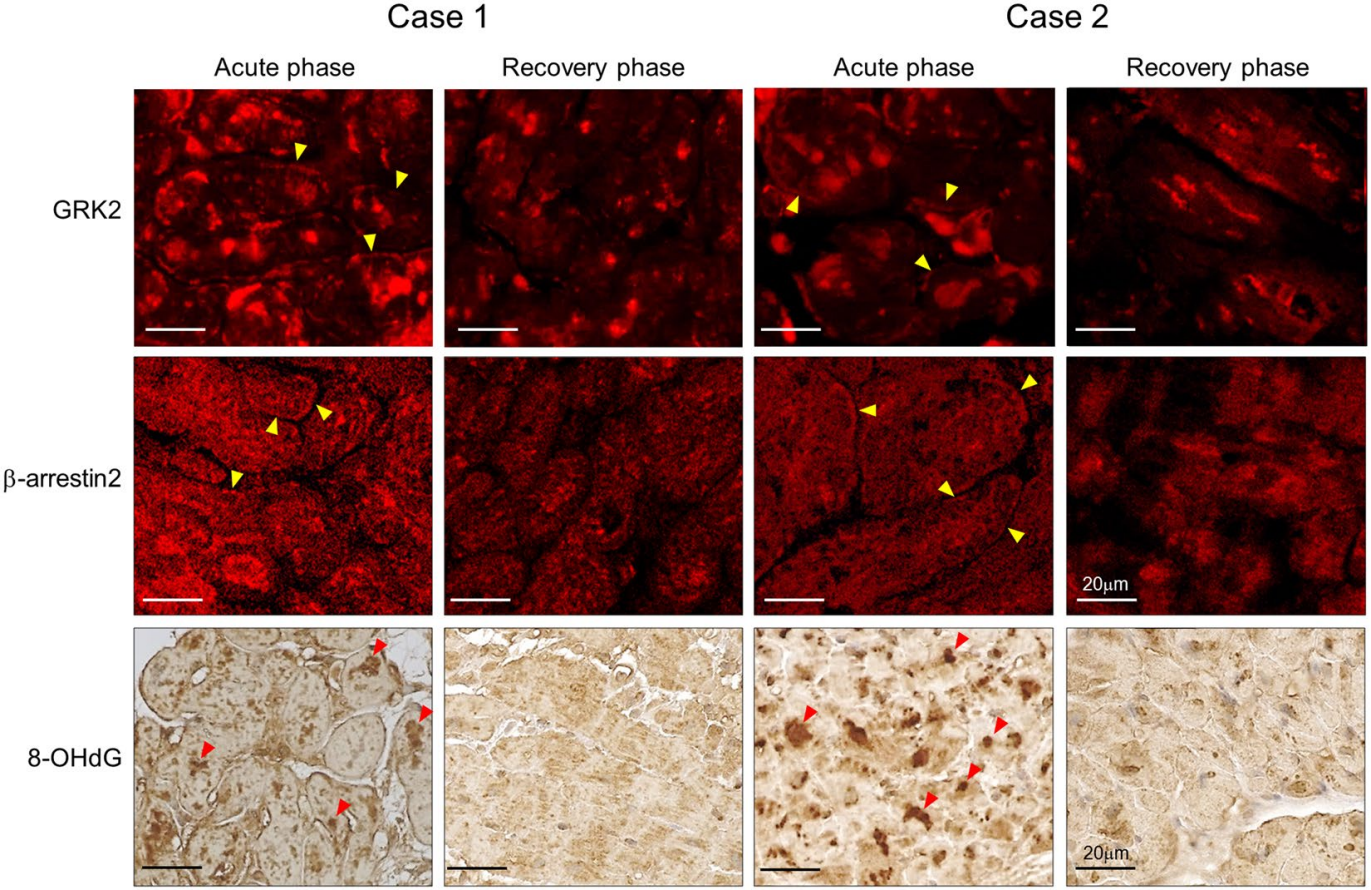
Localization of G protein-coupled receptor kinase 2 (GRK2), β-arrestin2, and 8-hydroxy-2′deoxyguanosine (8-OHdG) in two patients with takotsubo syndrome who underwent endomyocardial biopsy in both the acute and recovery phases. Micrographs showing immunofluorescence stainings of GRK2 and β-arrestin2, and immunohistochemical staining of 8-OHdG in the acute and recovery phases. Arrowheads (yellow) indicate positive signals for GRK2 and β-arrestin2 on the cell membrane, and Arrowheads (red) indicate 8-OHdG-positive nuclei.

Immunohistochemical stainings were also carried out for two patients with TTS in the recovery phase as shown in Fig. 4. One patient (Case 1) underwent the second LV endomyocardial biopsy on the 13^th^ day and the other (Case 2) on the 20^th^ day after admission, when LV wall motion and cardiac parameters had recovered to the normal range. In the recovery phase, the GRK2- and β-arrestin2-positive areas in the myocardium were only slightly reduced as compared to the acute phase, from 43% to 38% and from 45% to 38%, respectively, in Case 1, and from 30% to 27% and 44% to 38%, respectively, in Case 2. However, the fraction of cardiomyocytes positively stained for GRK2 and β-arrestin2 on the cell membrane was markedly reduced in the recovery phase from 15% to below the detection limit and from 11% to 1%, respectively, in Case 1, and from 34% to 7% and from 13% to 3%, respectively, in Case 2. Furthermore, the fractions of 8-OHdG-positive nuclei were substantially reduced in the recovery phase; from 27% to 10% in Case 1 and from 23% to 9% in Case 2.

## Discussion

While earlier reports assumed an association between SNS activation and TTS^1,5,10,12–14,20,23^, this had not been verified in human heart tissue to date. Using human LV biopsy samples from patients with TTS at the acute phase, the present immunohistochemical study demonstrated, to our knowledge for the first time, that GRK2 and β-arrestin2 are overexpressed in TTS cardiomyocytes and distributed throughout the cytoplasm, along with partial translocation to the cell membrane, which are initial steps of β-AR desensitization and subtype switching of the coupling G protein from Gαs to Gαi, leading to reduced LV contraction.

Alteration of β-AR signaling has been well studied in chronic heart failure^18,19^. In accordance with these studies, the present study revealed higher expression of GRK2 and β-arrestin2 in the LV myocardium in patients with DCM. This alteration of β-AR signaling, as indicated by GRK2 and β-arrestin2 overexpression in the LV myocardium, was also observed in acute-phase TTS. Notably, we observed not only higher GRK2 and β-arrestin2 expression in the cytoplasm, but the fraction of cardiomyocytes with membrane-positive staining for both molecules was higher in TTS than in DCM. These observations support the concept that β-ARs on the cell membrane are modified by GRK2 and β-arrestin2, which might play a key role in the development of LV dysfunction in TTS, which presumably prevents catecholamine-induced cardiac tissue damage via a trade-off between cell survival and reduced wall motion. Actually, signals of pCREB, which is phosphorylated by PKA and linked to cardiomyocyte contraction, were not increased in TTS, suggesting β-AR/PKA signaling pathway is not activated in TTS, while those were increased in DCM. Although the PKA signaling has been reported in experimental models of TTS, some reported it as activated as Borchert *et al*. analyzed in an induced pluripotent stem cells-derived cardiomyocyte obtained from TTS patient^23^, others reported it as deactivated as Cao *et al*. studied in an ovariectomized rat model of TTS^24^, which is still controversial.

In two patients, we performed a second LV endomyocardial biopsy in the recovery phase, when LV function had been recovered. Samples were obtained from the LV wall, in which wall motion had been severely reduced in the acute phase. In contrast to the acute phase, in the recovery phase, the fraction of cardiomyocytes with GRK2 or β-arrestin2 signal in the membrane was substantially decreased, while the signals in the cytoplasm were only marginally diminished. This serial change in expression profiles of GRK2 and β-arrestin2, especially on the cell membrane, was roughly associated with the decline and recovery of LV wall motion, which indicates the importance of membrane translocation of these molecules in the phenotype of TTS.

When comparing the extent of upregulation of GRK2 and β-arrestin2 expression between TTS and DCM, both molecules were higher expressed in the cytoplasm and cell membrane in acute-phase TTS than in DCM, which is apparently associated with the severer LV hypokinesis in TTS than in DCM. Although the underlying mechanism was not evaluated in the present study, this might reflect a higher and more rapid elevation of the catecholamine levels, termed “catecholamine surge”, in TTS^5^. In addition to the transient upregulation of GRK2 and β-arrestin2, their transient translocation to the cell membrane would be characteristic of TTS, and presumably reflects the strength of catecholamine stimulation immediately before biopsy and results in the transient, not persistent, hypokinesis of TTS hearts.

ROS production reportedly is a response to β-AR stimulation^25–27^ and is another factor in transient hypokinesis of the LV in TTS^25^. Therefore, we investigated the extent of ROS production by immunohistochemistry of 8-OHdG. Acute-phase TTS hearts showed significantly more 8-OHdG-positive cardiomyocyte nuclei than control and DCM hearts. In the recovery phase, the fraction of 8-OHdG-positive nuclei in TTS hearts was reduced to the level in control hearts. Although ROS are produced not solely in response to β-AR signaling, the expression pattern of 8-OHdG was similar to those of GRK2 and β-arrestin2 on the cell membrane, indicating that ROS might also be involved in altering β-AR signaling in TTS.

**Figure 5:**
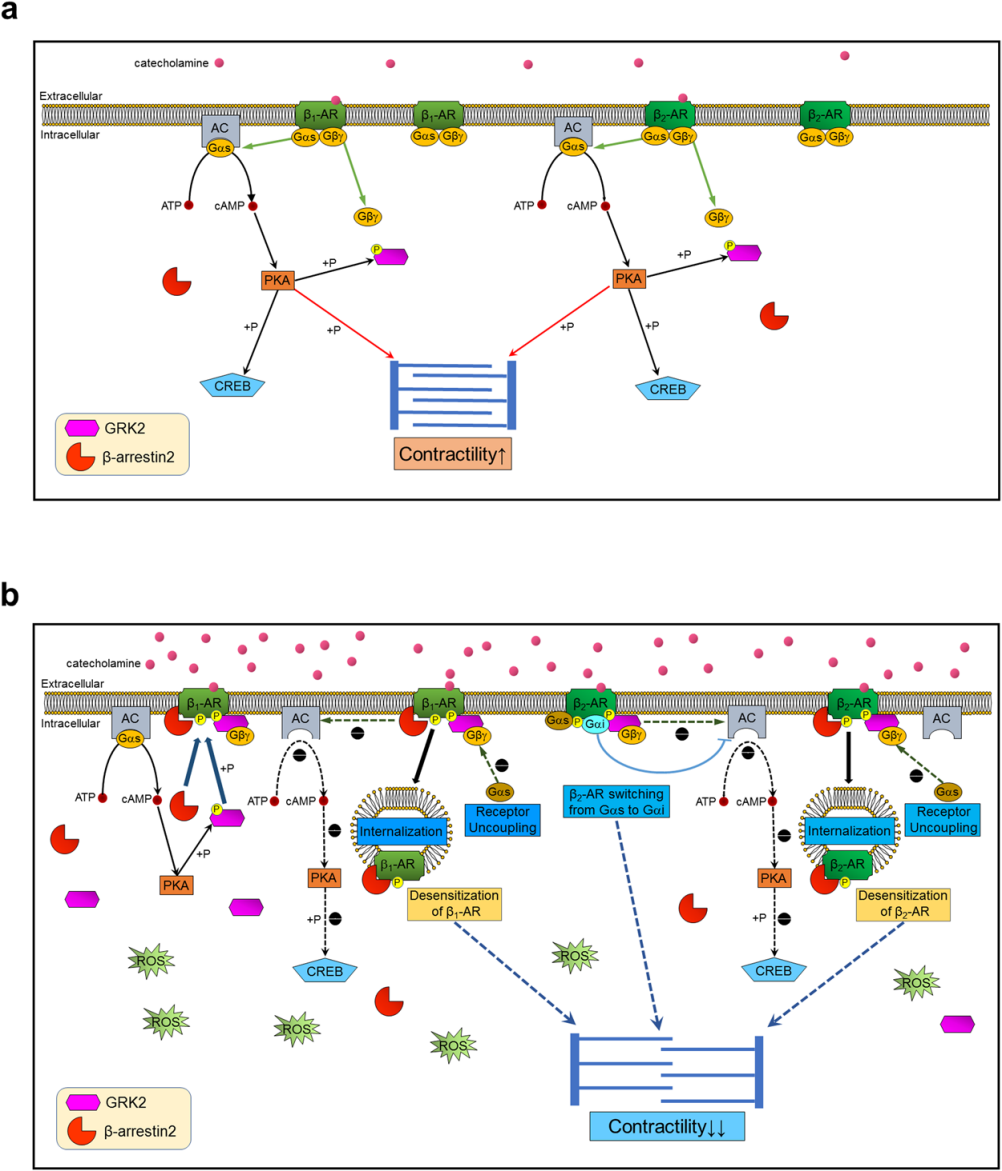
A proposed mechanism of takotsubo syndrome (TTS). (**a**) β-adrenoceptors (ARs) signaling including G protein-coupled receptor kinase 2 (GRK2) and β-arrestin2 under physiological catecholamine concentrations in cardiomyocyte, which mirrors the state in the normal control group. (**b**) β-ARs signaling under supraphysiological catecholamine concentrations in cardiomyocyte. GRK2 and β-arrestin2 are overexpressed and subsequently phosphorylation of cyclic-AMP response element binding protein (CREB) is decreased by the inactivation of protein kinase A (PKA) signaling. The reactive oxygen species (ROS) is also produced. GRK2 and β-arrestin2 translocate to the cell membrane, where they are thought to trigger receptor internalization, G-protein uncoupling in β_1_- and β_2_-ARs, and Gα protein switching from stimulatory Gαs to inhibitory Gαi in β_2_-AR. These protective reactions of β-ARs against catecholamine excess and ROS production are associated with some of the mechanisms of developing left ventricular hypokinesis in the TTS heart. AC: adenylyl cyclase, cAMP: cyclic adenosine monophosphate, ATP: adenosine triphosphate.

Based on our findings, we propose a model on how the studied factors are related in acute-phase TTS in Fig. 5.

The present findings, of course, cannot explain the hyperkinesis of the LV base, which is another characteristic of TTS. Recently, Lyon *et al*. proposed an interesting hypothesis that hyperkinesis in the LV base with apical hypokinesis may result from the higher tissue density of β-ARs in the apex than in the base^20^. Namely, the physiological range of catecholamine positively affects LV wall motion through β-ARs, while its supraphysiological elevation could prompt β-ARs desensitization, resulting in LV hypokinesis, especially in the apical portion. Moreover, the β_2_-AR positive inotropic effect is intercepted by switching its coupling G protein from Gαs to Gαi, which occurs in the presence of a supraphysiological catecholamine concentration, especially, adrenaline, because of the higher affinity of β_2_-AR to Gαi under elevated adrenaline concentration^18,19,28^. β_2_-AR-Gαi coupling also activates the phosphoinositide 3 kinase-protein kinase B (Akt) pathway, which exerts an anti-apoptotic effect^29,30^. Akt pathway activation in the acute-phase of TTS myocardium has been reported^29^. In this study, we preliminarily observed the down-regulation of β_1_-AR expression both in TTS and DCM, while the down-regulation of β_2_-AR was observed only in DCM. The expression of β_2_-AR in TTS was as normal level as in normal controls (data not shown). Thus, in the apical LV samples analyzed in this study, β_2_-AR expression may be relatively up-regulated compared with β_1_-AR expression, which partially supports the Lyon’s hypothesis of β-ARs distribution and the development of TTS^20^. Further studies are necessary to elucidate the precise expression profile of β_1_- and β_2_-ARs in human TTS patients.

Several studies have reported that the β_3_-AR may mediate a countervailing influence on β_1_ and β_2_-ARs, resulting in function as “physiological brake” to reduce the effect of sympathetic stimulation^31,32^. Given that β_3_-AR is not downregulated in heart failure^31–34^, it remains to be solved that β_3_-AR is involved in the pathogenesis of TTS.

There are some additional limitations to this study. Firstly, the sample size was small, and all samples were obtained from a single hospital. However, with respect to the histologic study of human biopsy samples, this study is larger than previous studies^1,29,35^. Secondly, only 2 samples were evaluated in the recovery phase. The sample size was too small to lead to any conclusion or perform a statistical calculation, thus larger number of samples is needed for the better understanding of the mechanisms of developing TTS. Thirdly, several papers reported the correlation between GRK2 or β-arrestin2 expression and aging^36–40^. In our study, although aging did not affect the expressions of GRK2 or β-arrestin2 in TTS (Data not shown), there might be some influence of aging on the result of increased expression of those molecules in TTS because the age was significantly older in the TTS group. Further investigation is needed to elucidate the relation between aging and the development of TTS. Fourthly, we conducted only immunohistochemistry for protein expression analysis. Generally, it is better to combine western blotting to confirm the result of immunohistochemistry because this technique depends on the specificity and sensitivity of the antibodies used, and is semi-quantitative. In this human study, we could not obtain large samples enough to extract protein for western blotting. To partly compensate for the limited methodology in this study, western blot analysis using autopsied human sample was performed, which showed a single band for each target molecule as shown in Supplementary Fig. S2.

In conclusion, we provided — to our knowledge for the first time — histologic evidence of the alteration of β-ARs via GRK2 and β-arrestin2 overexpression and their partial translocation to the cell membrane in the acute phase of TTS using biopsied human LV specimens. Transient catecholamine cardiotoxicities and subsequent β-AR’s cardioprotective alteration could be involved in the mechanism of TTS development.

## Methods

### Study population

Sixty-nine patients admitted to our hospital between January 2003 and November 2016 were diagnosed as TTS according to the Mayo Clinic criteria^41^. Among these 69 patients, 26 underwent LV endomyocardial biopsy in the acute phase to exclude myocarditis or other cardiomyopathies, and all of them were enrolled in this study (TTS group, for details, see Supplementary Table S2). As controls with normal LV function, 19 consecutive patients with conduction disorder referred to our hospital between January 2003 and November 2016, were enrolled (Normal Control: NC group). NC patients were revealed not to have coronary artery disease nor LV dysfunction by coronary angiography and left ventriculography, and underwent LV endomyocardial biopsy to exclude primary myocardial diseases. As controls with impaired LV function, 26 consecutive patients who were diagnosed clinically and confirmed pathologically by endomyocardial biopsy as DCM between January 2007 and October 2008 were enrolled (DCM group). Patients who had been administered β-receptor blocker before admission were excluded to evaluate the factors associated with β-ARs and their related molecules.

The study protocols were approved by the Nara Medical University Ethics Committee, #13-8, and was followed the 1975 Declaration of Helsinki guidelines. Informed consent was obtained from all participants and/or their legal guardians.

### Patient and histologic assessments

In all three groups, data from laboratory tests, left ventriculography, and transthoracic echocardiography were assessed. We assessed the LV function by left ventriculography in the acute phase and by transthoracic echocardiography before discharge.

Endomyocardial biopsy specimens were taken from the LV posterior wall in all patients. In the TTS group, two patients underwent endomyocardial biopsy twice during hospitalization, in the acute and recovery phases.

Biopsy specimens were processed for HE and MT stainings and immunohistochemistry. Interstitial fibrosis was quantified as a percentage of total myocardium by MT staining, using a BZ-X710 microscope and analyzer software system (BZ-X710 system, Keyence, Osaka, Japan). The endocardium and vascular structure were excluded from the total myocardium area.

For immunohistochemistry, anti-GRK2 (NBP2-37611, 1:200 dilution; Novus Biologicals, Littleton, CO), anti-β-arrestin2 (#3857, 1:50 dilution; Cell Signaling Technology, Danvers, MA), WGA-Alexa Fluor 488 (W11261, 1:2000 dilution; Invitrogen, Carlsbad, CA), anti-pCREB (Ser133) (#9198, 1:400 dilution; Cell Signaling Technology) and anti-8-OHdG (ab48508, 1:200 dilution; Abcam, Cambridge, United Kingdom) antibodies were used. Detailed methodology is available in the Data Supplement.

GRK2- and β-arrestin2-positive areas in the myocardium were assessed as percentages of total myocardium under the BZ-X710 system. For this quantification, the endocardium, vascular structure, and interstitial tissue were excluded from the myocardium area, and patchy staining area — mainly owing to lipofuscin — was excluded from the positively stained area. To assess GRK2- or β-arrestin2-positive signal on the cell membrane, we counted the number of cardiomyocytes in which GRK2 or β-arrestin2 signal colocalized with WGA, a cell membrane marker, and calculated the proportion of positively stained cardiomyocytes as a percentage of the total number of cardiomyocytes.

pCREB-positive and 8-OHdG-positive nuclei in cardiomyocytes were evaluated as a percentage of the total number of cardiomyocyte nuclei, using the BZ-X710 system. These histologic evaluations were carried out by three independent expert cardiologists who were blinded to all clinical data.

### Statistical analysis

Continuous variables are expressed as means and standard deviations or medians (25^th^–75^th^ percentile) as appropriate, and categorical variables are expressed as percentages. Statistical significance of continuous variables between three groups was assessed with one-way analysis of variance or the Kruskal-Wallis test, followed by the Tukey honest significant difference test or the Mann-Whitney test, as appropriate. Statistical significance of categorical variables was assessed by chi-square analysis. P-values of less than 0.05 were considered statistically significant. JMP software for Windows version 12 (SAS Institute, Cary, NC) was used to analyze data.

## Electronic supplementary material

Supplementary Appendix

## Data Availability

No datasets were generated or analysed during the current study.
