# Supplementary material for: Alteration of β-Adrenoceptor Signaling in Left Ventricle of Acute Phase Takotsubo Syndrome: a Human Study
Source: Sci Rep. 2018 Aug 24;8:12731. doi: 10.1038/s41598-018-31034-z (PMC6109068; doi:10.1038/s41598-018-31034-z)
Supplement: Supplementary file 1 — Supplementary Appendix [file 41598_2018_31034_MOESM1_ESM.pdf]

Supplementary Information

Alteration of β-Adrenoceptor Signaling in Left Ventricle of Acute Phase Takotsubo Syndrome: a Human Study

Tomoya Nakano[1], Kenji Onoue[1]*, Yasuki Nakada[1], Hitoshi Nakagawa[1], Takuya Kumazawa[1], Tomoya Ueda[1], Taku Nishida[1], Tsunenari Soeda[1], Satoshi Okayama[1], Makoto Watanabe[1], Hiroyuki Kawata[1], Rika Kawakami[1], Manabu Horii[1,3], Hiroyuki Okura[1], Shiro Uemura[1,4], Kinta Hatakeyama[2], Yasuhiro Sakaguchi[1] & Yoshihiko Saito[1]

[1] First Department of Internal Medicine, Nara Medical University, Kashihara Nara, Japan

[2] Department of Diagnostic Pathology, Nara Medical University, Kashihara Nara, Japan

[3] Current affiliation; Department of Cardiology, Nara City Hospital, Nara, Japan

[4] Current affiliation; Department of Cardiology, Kawasaki Medical School, Kurashiki, Japan

*Corresponding author.

Supplemental Methods

Immunohistochemistry protocol using endomyocardial biopsy specimens

The biopsied specimens were fixed with 10% buffered formalin, embedded in paraffin, and sectioned at 3-μm thickness. After deparaffinization, the tissues were blocked with Tris-buffered saline with Tween-20 (TBST) containing 4% normal donkey Serum (Sigma-Aldrich, St. Louis, MO) and 2% bovine serum albumin for 1 hour. Then, they were incubated with primary antibodies at 4°C overnight. For G protein-coupled receptor kinase 2 (GRK2) immunostaining, goat anti-mouse IgG-Alexa Fluor 647 (A-21235, 1:500 dilution; Invitrogen, Carlsbad, CA) was used as the secondary antibody and wheat germ agglutinin (WGA)-Alexa Fluor 488 (W11261, 1:2000 dilution; Invitrogen) was used to detect the cell membrane. Similarly, for β-arrestin2 immunostaining, goat anti-rabbit IgG-Alexa Fluor 647 (A-21245, 1:500 dilution; Invitrogen) was used as the secondary antibody. The tissues were mounted with ProLong Gold antifade reagent with 4’6-diamidino-2-phenylindole (DAPI) (Molecular Probes, Eugene, OR). For 8-hydroxy-2’-deoxyguanosine (8-OHdG) immunostaining, Histofine Simple Stain Max PO (M) (424131, Nichirei, Tokyo, Japan) was used as the secondary antibody and diaminobenzidine (Simple Stain DAB Solution, 425011, Nichirei) was used as a chromogenic substrate. Similarly, Phosphorylation of cyclic-adenosine monophosphate response element binding protein at Ser133 (pCREB (Ser133)), Histofine Simple Stain Max PO (R) (424141, Nichirei, Tokyo, Japan) was used as the secondary antibody. All sections were counterstained

with hematoxylin. As a negative control, tissues were stained using normal rabbit or mouse IgG instead of the specific primary antibody as shown in Supplementary Fig. S4.

Western blot analysis protocol using autopsied human cardiac sample

Autopsied human cardiac sample was lysed in tissue protein extraction reagent (Thermo Scientific, Rockford, IL) with complete EDTA-free protease inhibitor (Roche Life Science, Mannheim, Germany) and Halt Phosphatase Inhibitor Cocktail (Thermo Scientific). Equal amounts of protein for each sample were separated by sodium dodecyl sulfate polyacrylamide gel electrophoresis and transferred onto a polyvinylidene difluoride membrane. The membrane was blocked for 1 hour in TBST containing milk powder. The membrane was incubated with primary anti-GRK2 antibody (NBP2-37611, 1:2000 dilution; Novus Biologicals) and anti-β-arrestin2 antibody (#3857, 1:1000 dilution; Cell Signaling Technology) at 4°C overnight, and then with secondary HRP-conjugated antibody (1:5000 dilution, Invitrogen) at room temperature for 1 hour. Two milliliters of Western Lightning Plus-ECL chemiluminescence detection kit (Perkin-Elmer, Waltham, MA) was used for protein detection. Glyceraldehyde-3-phosphate dehydrogenase (GAPDH) (M171-3, 1:5000 dilution; Medical & Biological Laboratories Co., Nagoya, Japan) was used as a loading control.

Supplemental Results

Supplementary Table S1. Transthoracic echocardiographic data before discharge.

	**Normal control (n = 19)**	**Takotsubo syndrome (n = 26)**	**Dilated cardiomyopathy (n = 26)**
Interventricular septum thickness (mm)	9.8 ± 1.2	10.6 ± 1.9	10.0 ± 2.2
Posterior wall thickness (mm)	9.6 ± 1.2	10.5 ± 1.7	10.0 ± 1.9
LV end-diastolic dimension index (mm/m^2)	28.2 ± 3.2	30.5 ± 4.3	36.3 ± 4.8 ‡
LV end-systolic dimension index (mm/m^2)	17.4 ± 3.1	19.3 ± 3.9	30.3 ± 4.1 ‡
LV ejection fraction (%) *	67.4 ± 7.0	62.2 ± 13.2	33.7 ± 6.0 ‡
E/A	1.1 ± 0.4	0.8 ± 0.3 †	1.2 ± 0.6 §
Left atrial dimension index (mm/m^2)	21.0 ± 3.0	25.4 ± 4.1 †	25.9 ± 5.6

Values are mean ± standard deviation. * LV ejection fraction was calculated using the modified biplane Simpson method. † P < 0.001 vs. Normal control. ‡P < 0.001 vs. Takotsubo syndrome. §P < 0.01 vs. Takotsubo syndrome. LV: left ventricle.

Supplementary Table S2. Clinical Characteristics of 26 patients with takotsubo syndrome

Patient No.	Age	Sex	Stressor	Clinical Presentation on Admission		
				Time after symptom onset - hour *	Symptoms	Type of takotsubo syndrome†
1	68	F	Fear of surgery	1	Cardiac shock	Midventricular Type
2	83	F	Death of sister	1	Heart failure	Apical Type
3	79	F	Argument with family	2	Chest pain	Apical Type
4	86	F	Taking care of spouse	2	Chest pain	Apical Type
5	67	M	Fire	2	Heart failure	Focal Type
6	89	F	Fear of transesophageal echocardiography	3	Chest pain, Heart failure	Apical Type
7	46	F	Hyperthyroidism	3	Dyspnea	Apical Type

8	76	F	None	3	Chest pain	Apical Type
9	72	F	Argument with neighbor	4	Chest pain	Apical Type
10	54	F	Argument with family	4	Chest pain	Apical Type
11	81	F	Depression	4	Chest pain	Midventricular Type
12	69	F	None	5	Chest pain	Apical Type
13	76	F	Death of cousin	6	Chest pain	Apical Type
14	71	M	Depression	6	Dyspnea	Apical Type
15	76	F	Argument with neighbor	6	Chest pain	Apical Type
16	90	F	None	7	Chest pain	Apical Type
17	40	F	None	10	Heart failure	Apical Type
18	75	M	Pneumonia	12	Chest pain	Apical Type
19	74	M	None	14	Chest pain	Apical Type

20	73	F	Argument with neighbor	22	Chest pain	Apical Type
21	76	F	None	22	Heart failure	Apical Type
22	71	F	Diving	36	Chest pain	Apical Type
23	56	F	None	48	Chest pain	Apical Type
24	72	F	Fracture	48	Heart failure	Apical Type
25	79	F	Death of pet	58	Dyspnea	Apical Type
26	75	F	Death of spouse	72	Chest pain	Apical Type

*Values are times from the onset of symptoms to admission. The median was 6.0 (3.0-22.0) hours.

†Based on LV angiography, takotsubo syndrome was classified into one of four types: apical, midventricular, basal, and focal[1].

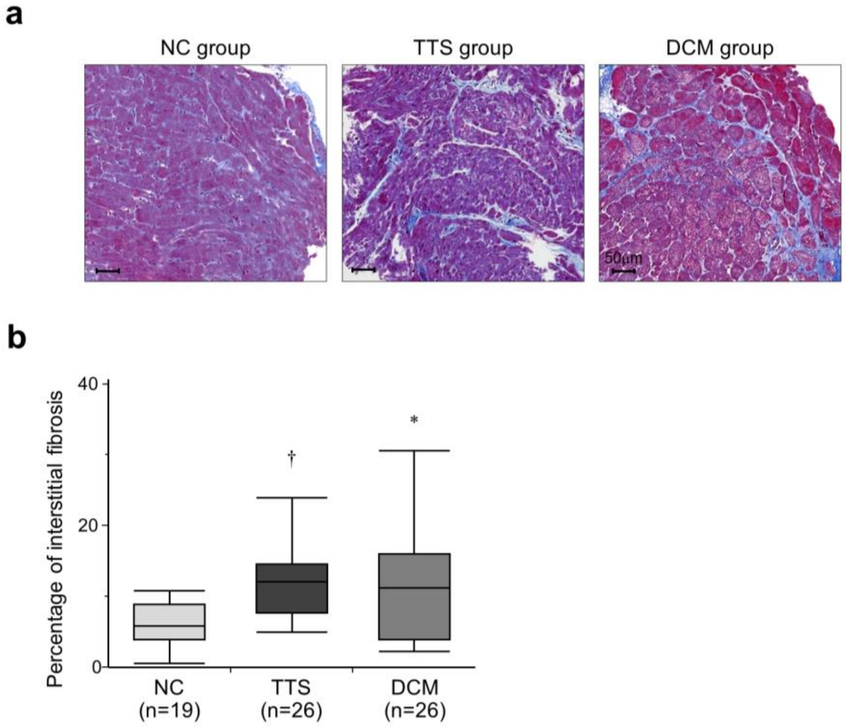

Supplementary Figure S1. Interstitial fibrosis. **(a)** Micrographs show Masson's trichrome (MT) staining for interstitial fibrosis (blue). **(b)** The bar graph shows the quantification of interstitial fibrosis as a percentage of total myocardium, based on MT staining. The box represents the 25th and 75th percentiles and the line the median value. Whiskers correspond to the 25th percentile minus 1.5 times interquartile range (IQR) and to the 75th percentile plus 1.5 IQR. *P < 0.05 vs. the normal control (NC) group. †P < 0.001 vs. the NC group. DCM: dilated cardiomyopathy, TTS: takotsubo syndrome.

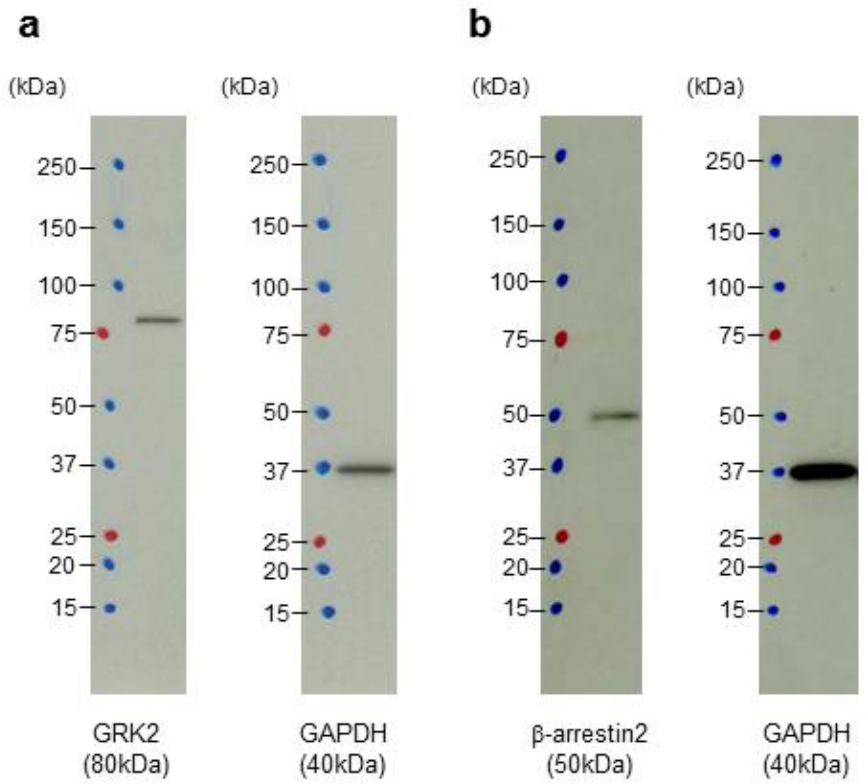

Supplementary Figure S2. Western blot analysis for anti-G protein-coupled receptor kinase 2 (GRK2) and anti-β-arrestin2 antibodies. To evaluate the specificity of the antibodies used for GRK2 and β-arrestin2 immunostainings, western blot analysis was performed using the conventional method and human cardiac tissue from an autopsied patient. Both anti-GRK2 **(a)** and anti-β-arrestin2 **(b)** antibodies showed a single band. The pictures show full length blots without cropping after development. Color dots of blue and red indicate the loading marker. GAPDH: glyceraldehyde-3-phosphate dehydrogenase.

a

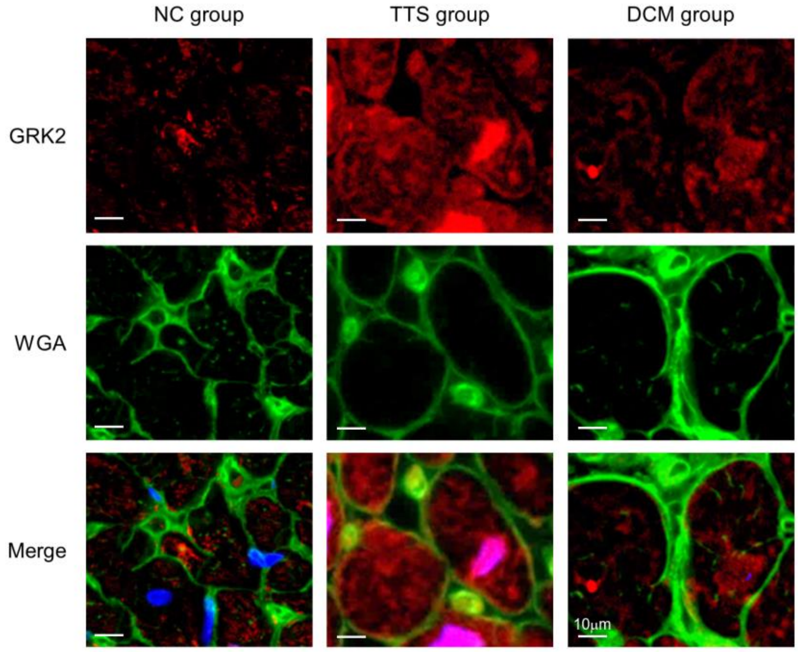

b

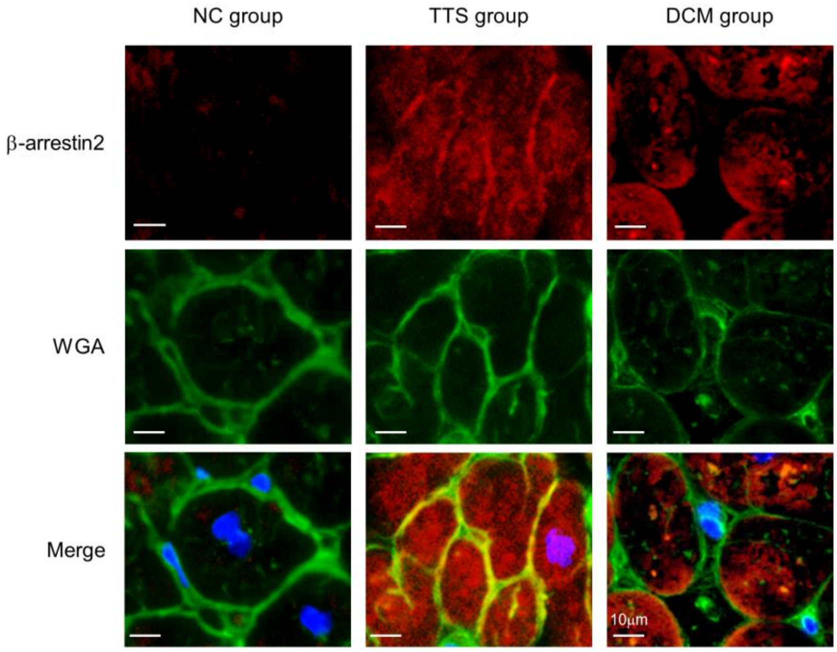

Supplementary Figure S3. Enlarged images of GRK2 and β-arrestin2 immunostaining. **(a)** GRK2 immunostaining. **(b)** β-arrestin2 immunostaining.

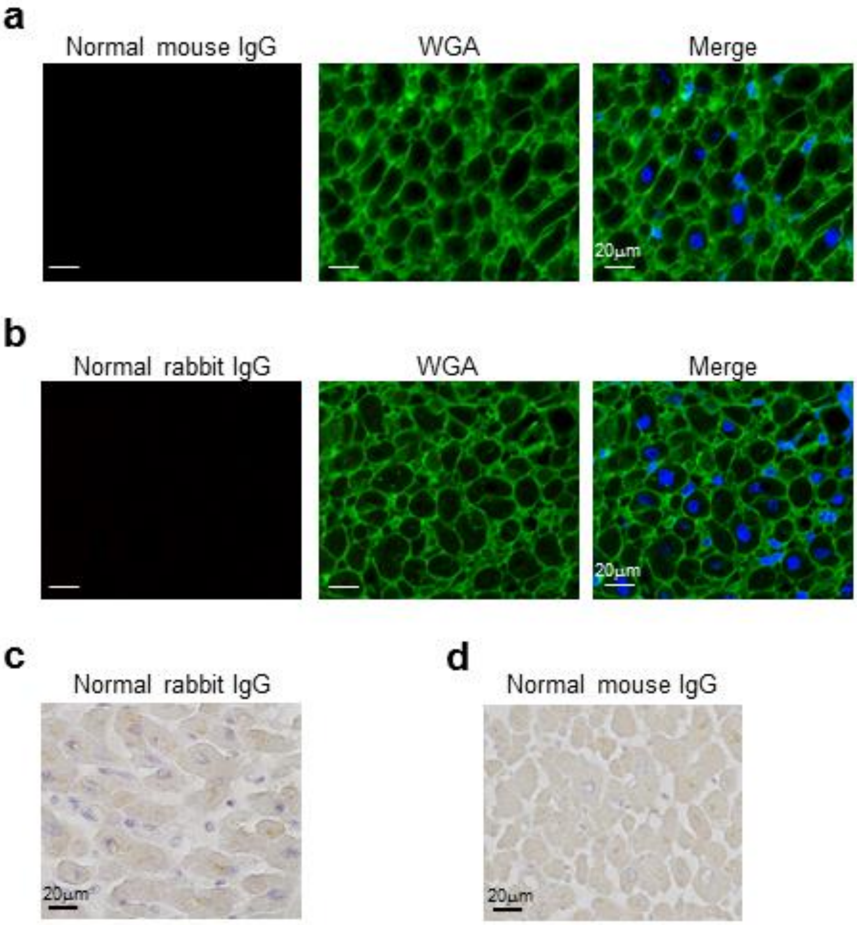

Supplementary Figure S4. Negative control of immunohistochemistry. **(a)** Normal mouse IgG instead of anti-G protein-coupled receptor kinase 2 (GRK2) antibody was used as a negative control for GRK2 immunostaining. Secondary antibody and wheat germ agglutinin (WGA) (green) were used according to immunohistochemistry protocol. Blue staining localized to 4',6-diamidino-2-phenylindole (DAPI). **(b)** Normal rabbit IgG instead of anti-β-arrestin2 antibody was used as a negative control for β-arrestin2 immunostaining. Secondary antibody and WGA (green) were used according to immunohistochemistry protocol. Blue staining localized to DAPI. **(c)** Normal rabbit IgG instead of anti-phosphorylated cyclic-AMP response element binding protein at Ser133 (pCREB (Ser133)) antibody was used as a negative control for pCREB immunostaining. **(d)** Normal mouse

IgG instead of anti-8-hydroxy-2’-deoxyguanosine (8-OHdG) antibody was used as a negative control for 8-OHdG immunostaining.

Supplemental Reference

1. Templin, C. *et al*. Clinical features and outcomes of Takotsubo (stress) cardiomyopathy. *N Engl J Med.* **373,** 929-38, doi: 10.1056/NEJMoa1406761 (2015).
